# Recent Advancements in 2D Material-Based Memristor Technology Toward Neuromorphic Computing

**DOI:** 10.3390/mi15121451

**Published:** 2024-11-29

**Authors:** Sungmin Park, Muhammad Naqi, Namgyu Lee, Suyoung Park, Seongin Hong, Byeong Hyeon Lee

**Affiliations:** 1 Department of Physics, Gachon University, Seongnam 13120, Republic of Korea; 2 Department of Electronic Engineering, University of Exeter, Exeter EX4 4QF, UK; 3 Department of Semiconductor Engineering, Gachon University, Seongnam 13120, Republic of Korea; 4 Department of Microdevice Engineering, Korea University, Seoul 02841, Republic of Korea

**Keywords:** 2D materials, memristor, neuromorphic, in-memory computing, artificial synapse

## Abstract

Two-dimensional (2D) layered materials have recently gained significant attention and have been extensively studied for their potential applications in neuromorphic computing, where they are used to mimic the functions of the human brain. Their unique properties, including atomic-level thickness, exceptional mechanical stability, and tunable optical and electrical characteristics, make them highly versatile for a wide range of applications. In this review, we offer a comprehensive analysis of 2D material-based memristors. Furthermore, we examine the ability of 2D material-based memristors to successfully mimic the human brain by referencing their neuromorphic applications.

## 1. Introduction

In the last decade, the “big data” era has emerged as an active research and development hotspot in technologies capable of processing massive datasets driven by rapid technological advancements. Emerging technologies, such as artificial intelligence (AI), the Internet of Things (IoT), and virtual and augmented reality, leverage this wealth of information. Von Neumann proposed an architecture based on metal–oxide–semiconductor field-effect transistors featuring separation and linear interactions. However, current structures face inefficiencies such as data bottlenecks and high power consumption when processing extensive datasets. Exploring new structures and materials to overcome these challenges has gained significant interest. In this regard, neuromorphic computing, inspired by the structure of the human brain, has gained considerable attention. Efforts are underway to process vast amounts of information with minimal power consumption by utilizing artificial neural network systems comprising neurons and synapses [1].

Research on neuromorphic computing encompasses various hardware and software approaches. One such approach is oscillator-based neuromorphic computing, which mimics the nonlinear operations of the brain by utilizing the dynamics of phase and frequency through coupled nano-oscillators [2]. Another emerging paradigm is wetware-based neuromorphic computing, which replicates the physicochemical information processing of biological systems using fluid solutions [3]. This approach leverages chemical AI in wetware, incorporating chemical reaction networks, synthetic biology, and nanofluidic iontronics to enable advanced neuromorphic functionality. Neuromorphic systems operate through “in-memory computing,” which enables the simultaneous processing and storage of data, akin to the function of neurons in the brain, which handle both memory storage and data processing [4,5,6]. Neuromorphic computing, which mimics the brain’s neural networks, can be performed with next-generation random access memory (RAM), which includes in-memory computing. There are two important aspects to mimic biological synapses: (1) device structure and (2) active material. The structural imitation method consists of two- and three-terminal structures, which generally have a three-terminal structure that utilizes a method driven through a separate gate terminal by forming a functional film such as a ferroelectric, ferromagnetic, or charge trap layer in the existing transistor structure. This structure can fine-tune the conductivity through separate control terminals; however, the additional process and the large size of the device increase its complexity. In contrast, two-terminal devices, which more closely resemble biological synapses, can be easily implemented using intermediate insertion layers such as those found in phase-change RAM [7,8], magnetoresistive RAM [9,10], and resistive RAM (RRAM) structures. These structures offer a high integration density and are particularly well suited for the imitation of artificial neural networks.

In this context, RRAM is particularly notable because of its superior performance metrics, such as an ultra-low power consumption of 2.7 pW [11], an on/off ratio of 10^7^ [12], retention times exceeding 10^4^ s [13], and endurance cycles exceeding 10^12^ [14]. RRAM devices are typically constructed using a metal–insulator–metal (MIM) structure, exhibit resistive switching (RS) behavior within the insulator layer. This enables transitions between high-resistance states (HRSs) and low-resistance states (LRSs) through mechanisms such as vacancy migration, filament formation, or phase transitions, among others. The performance characteristics of RRAM devices are highly dependent on the choice of materials used for the insulator layer and electrodes [15]. Various materials, including perovskites [16,17], organics [12,13], solution-processed materials [18], metal oxides [14,18,19], and two-dimensional (2D) materials [20,21], have been explored for fabricating RRAM devices. Notably, RRAM based on two-dimensional (2D) materials regulates resistance states through atomic-level defects [22,23]. The defect density and distribution in 2D material-based RRAM significantly influence its performance, offering a high on/off ratio, rapid switching speed, and superior energy efficiency compared to conventional MIM structures [24]. Therefore, 2D materials are particularly noteworthy because of their ability to precisely control ultrathin atomic features and their unique electronic and mechanical properties, such as interfacial charge trapping, ferromagnetism, ferroelectricity, polymorphism, and phase transformations [25]. By stacking different materials or incorporating 2D materials, various heterogeneous structures with diverse electrical and optical properties can be created. These structures can be used to develop highly integrated artificial neural networks in crossbar array devices. The exceptional thinness of these materials is ideal for heterojunction structures, overcoming the single-channel effect, enabling fast electrical switching in memristor devices, and maximizing energy efficiency.

In this review, we provide a comprehensive overview of memory devices based on 2D materials, with a focus on their implementation in memristor designs and their various applications. The discussion is broadly divided into original and hybrid RRAM devices, based on the presence or absence of transformation or deposition processes. Particular emphasis is placed on RS mechanisms and the performance of devices as a function of the materials used in the RS layer. The final section of this review explores the applications of RRAM technology, with a focus on its potential for neuromorphic computing.

## 2. Two-Dimensional Material-Based Memristors: A Promise to Innovation

### 2.1. Mechanism of 2D Material-Based Memristors

**Figure 1:**
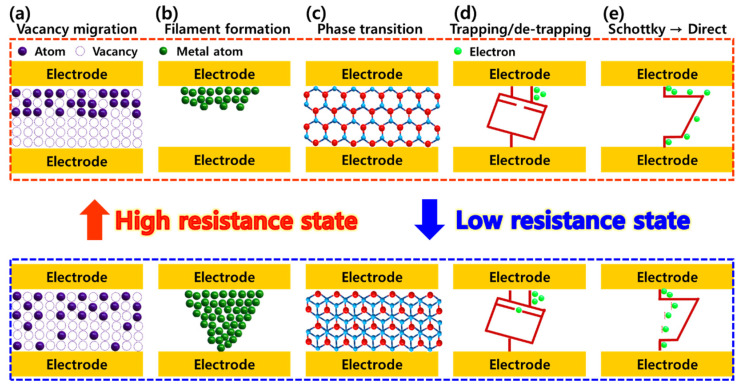
Schematic illustration of the operation mechanisms in vertical-structured, 2D material-based memristor devices. (**a**) Vacancy migration, (**b**) filament formation, (**c**) phase transition, (**d**) trapping/de-trapping, and (**e**) Schottky emission and direct tunneling.

The implementation of conventional memristors involves the use of 2D materials. These devices are typically designed as two-terminal structures with a vertically stacked configuration. Recently, advancements have expanded beyond the memristive layer to include upper and lower electrodes composed of graphene and other 2D materials [26]. The operational mechanism of such vertical structures varies depending on the hybrid type, in contrast to single 2D material cases. Figure 1 illustrates the operational mechanism of a memristor device with a vertical structure. When the active layers consist of only 2D materials, the proposed memristive switching mechanisms include filament formation [27,28], vacancy migration [29,30], phase transition [31], charge trapping/de-trapping [32], and transport conversion from Schottky emission to direct tunneling [33]. In contrast, memristor devices with hybrid structures, such as heterojunctions with 2D–2D or 2D–3D configurations, primarily operate via vacancy migration, filament formation, phase transition, and charge trapping/de-trapping, deviating from the mechanisms observed in previous 2D-only setups.

Chalcogen atoms, having relatively lower binding energy compared to transition metal atoms, can form vacancies due to thermodynamic factors, synthesis and deposition processes, or oxidation [34,35]. Figure 1a illustrates the vacancy migration model, where intrinsic vacancies formed under these conditions in certain 2D transition metal dichalcogenide (TMDC) materials are further influenced by external stimuli, such as electric fields. This contributes to the migration mechanism, ultimately affecting the resistance state. Filament formation, shown in Figure 1b, is a common driving mechanism in conventional resistive-switching memory devices. This mechanism involves the controlled formation of conductive filaments through external voltage sweeps. Typically, these filaments are created by ions from the electrode materials in the upper and lower parts and are driven by an external electric field. The LRS state is maintained in filament form and disintegrates when a reverse bias is applied, transitioning to the HRS. The characteristic phase transition behavior of 2D TMDC materials, as demonstrated in Figure 1c, using molybdenum disulfide (MoS_2_), reveals the potential for inducing the HRS and LRS by altering the electrical properties based on crystal structure deformations. For the resistance switching mechanism related to charge trapping and de-trapping, Figure 1d illustrates the behavior of charge generation at the interface between the electrode layer and the 2D TMDC layer. A positive voltage application leads to electron trapping at the interface, manifesting LRS characteristics, whereas a negative voltage application results in electron de-trapping, creating the HRS. Finally, Figure 1e depicts the resistive switching properties, where the mechanism shifts from Schottky emission to direct tunneling based on the stable structure of the 2D TMDC material and changes in the conduction properties due to temperature or external electric fields.

### 2.2. Various 2D Material-Based Memristor Devices

#### 2.2.1. α-In_2_Se_3_-Based Memristor

**Figure 2:**
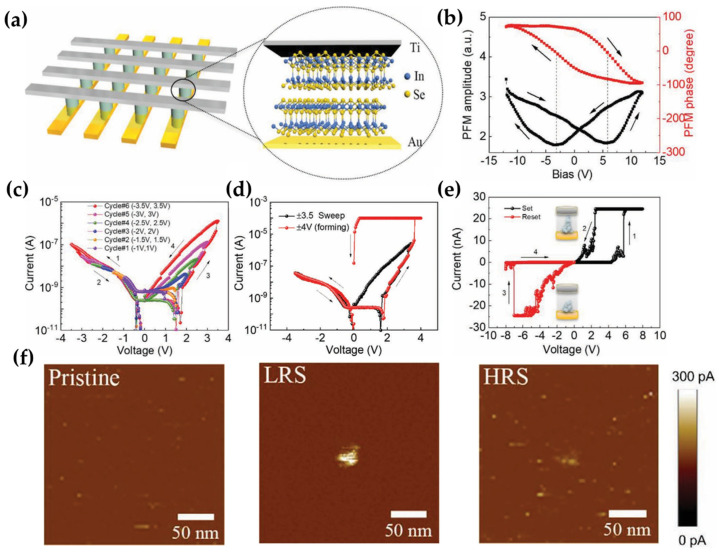
α−In_2_Se_3_−based RRAM memristor with analog and digital RS modes. (**a**) Schematic of the crossbar array structure of α−In_2_Se_3_−based memristors alongside a single memristor [36]. (**b**) Piezoresponse force microscopy results: amplitude and phase as a function of DC bias [36]. (**c**) Hysteresis loops with sweep voltages ranging from ±1 V to ±3.5 V [36]. (**d**) Forming process in digital mode at ±4 V [36]. (**e**) I–V curve in digital mode obtained via conductive atomic force microscopy (C−AFM) [36]. (**f**) C−AFM images of the device in its pristine, LRS, and HRS [36].

Zhang et al. introduced a memristor device based on 2H indium selenide (α-In_2_Se_3_) [36], utilizing a vertical crossbar structure capable of both digital and analog memory operations [37]. The device comprised a crossbar array structure featuring top electrodes (Ti), bottom electrodes (Au), and 2D α-In_2_Se_3_ positioned at each intersection (see Figure 2a). α-In_2_Se_3_ was the insulator in the MIM configuration of the memristor [38]. The dielectric properties of α-In_2_Se_3_ were verified, exhibiting out-of-plane ferroelectric polarization with a clear hysteresis loop in amplitude and phase versus DC bias (refer to Figure 2b). This device is distinguished by its ability to operate in analog and digital modes. RS in the analog mode was achieved through a ferroelectric polarization-controlled Schottky barrier mechanism. Using the Schottky emission model, expressed as $J=A\times T^{2}\exp\left[\frac{-q(\varphi -\sqrt{qE/4\pi \varepsilon )}}{KT}\right]$, where J is the current density, A is the Richardson constant, T is the absolute temperature, q is the electronic charge, φ is the Schottky barrier height, E is the electric field, ε is the dielectric constant, and K is the Boltzmann constant, V^1/2^-ln(I) exhibited a linear relationship, confirming that the RS behavior in this device is attributable to Schottky barrier modulation [39,40]. The analog RS mode manifested at a low V_sweep_, with a discernible minor hysteresis loop observed when V_sweep_ exceeded 1.5 V (refer to Figure 2c). This implies that the coercive voltage of α-In_2_Se_3_ stands at approximately 1.5 V.

The analog mode demonstrated robust performance metrics, including 5000 s of retention, over 100 cycles of reproducibility, and an on/off ratio of 20. Notably, the device demonstrated impressive performance in neuromorphic computing, a subject that will be examined in detail in a dedicated chapter along with other related devices. Interestingly, a digital mode was observed serendipitously. Applying V_sweep_ at 4 V resulted in an I–V curve distinct from that of the analog mode (Figure 2d), indicating a transition to the digital RS mode. The characteristic forming process, depicted in Figure 2e, is indicative of filament mechanism behavior, a phenomenon commonly associated with oxide−based RRAM but is uniquely observed in α−In_2_Se_3_. Given the inert nature of the Au/Ti electrodes, filament formation was speculated to occur in the Se vacancies. Conductive atomic force microscopy (C−AFM) was used to investigate whether the operation involved a filament-switching mechanism. Prior to the V_sweep_ application, no alterations were observed (pristine in Figure 2f). Subsequently, when V_sweep_ surpassed 6 V, an electric current jump occurred (black lines 1 and 2 in Figure 2e), accompanied by a bright region in the C−AFM image (LRS in Figure 2f). Upon exceeding −7.2 V during V_sweep_, the current decreased (red lines 3 and 4 in Figure 2e), and the bright region vanished (HRS in Figure 2f). This test confirmed the formation and rupture of filaments in the digital mode. Despite this, the transition from digital to analog mode proved to be challenging, owing to residual filaments. The two−terminal α−In_2_Se_3_−based memristor device exhibits greater compatibility with downsizing, occupying a smaller footprint, proving that it is more suitable for smaller crossbar structures. The study demonstrated that the α−In_2_Se_3_−based memristor can serve memory functions in digital mode and contribute to neuromorphic computing in analog mode.

#### 2.2.2. SnSe-Based Memristor

**Figure 3:**
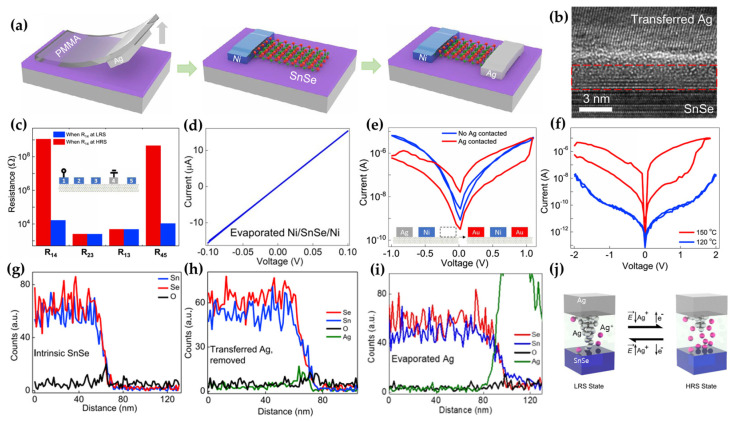
Ag/SnSe/Ni−based memristor. (**a**) Schematic of the fabrication process for the Ag/SnSe/Ni memristor [41]. (**b**) Cross−sectional transmission electron microscopy image of the Ag/SeO_x_/SnSe layer, with the SeO_x_ layer highlighted in the red dashed rectangle [41]. (**c**) Resistance measurements between five electrodes, comprising four Ni electrodes (1, 2, 3, and 5) and one Ag electrode (4), as shown in the inset [41]. (**d**) I–V curve of the Ni/SnSe/Ni device. (**e**) I–V curves for Ag−contacted and non−Ag−contacted devices [41]. (**f**) I–V curves of the memristor at annealing temperatures of 150 °C and 120 °C [41]. (**g**) Energy−dispersive spectroscopy (EDS) line−scan profile at intrinsic SnSe, (**h**) after the transfer−and−removal of the Ag process, and (**i**) with evaporated Ag [41]. (**j**) Schematic of the resistive switching (RS) mechanism in the Ag/SnO_x_/SnSe layer [41].

Guo et al. fabricated a Ni/tin selenide (SnSe)/Ag memristor via van der Waals (vdW) metal integration [41,42,43,44]. First, Ag was patterned onto a silicon oxide (SiO)/Si substrate and removed by sticking it to polymethylmethacrylate (PMMA). This process was necessary to prevent damage to the memristor during the Ag deposition stage. The Ni/SnSe was then deposited on a SiO_2_/Si substrate, followed by vertical Ag deposition opposite the Ni using vdW metal integration (Figure 3a). Conventional metal deposition methods, such as thermal evaporation and electron beam evaporation, damage the memristor and fail to achieve the desired performance. However, through Ag/SnSe deposition by vdW integration, native SnO_x_ on the surface of the intrinsic SnSe is preserved with only minimal damage [45,46]. Thus, an Ag/SnO_x_/SnSe vertical structure was formed such that the SnO_x_ and SnSe layers served as the insulator layer and channel, respectively (Figure 3b). In particular, the RS behavior of this memristor did not manifest between the top and bottom electrodes. The RS behavior of the memristor on the Ag/SnO_x_/SnSe layer was investigated. To confirm this, they checked the resistance of the connection between each of the four Ni electrodes (blue box) and one Ag electrode (gray box) on a SnSe flake (Figure 3c). In Figure 2c, R_nm_ denotes the resistance between electrodes n and m at the inset. R_14_ and R_45_ represent the resistances between the Ni and Ag electrodes, and R_23_ and R_13_ represent the resistances between only the Ni electrodes. R_14_ and R_45_ showed the HRS and LRS, whereas R_13_ and R_23_ showed only the LRS without the HRS. Thus, it was confirmed that the RS behavior occurred only in the Ag/SnO_x_/SnSe layer. In addition, the absence of RS behavior in R_13_ and R_23_ and the linear I–V curve of the Ni/SnSe/Ni device (Figure 3d) indicated that an ohmic contact was formed between Ni and SnSe. The mechanism underlying the RS behavior in the Ag/SnO_x_/SnSe layer was further investigated. In the experimental setup, Ag and Ni electrodes were deposited on the left and center of the SnSe, respectively, with no electrode on the right side. After annealing at 180 °C for 2 min, the Ag electrode on the left side was mechanically removed, and Au electrodes were deposited on the right and left sides where the Ag was removed (inset of Figure 3e).

They examined the I–V curves of the left and right Au/Ni. In Figure 3e, the I–V curve of the left Au/Ni shows hysteresis and RS behavior. In contrast, the I–V curve of the right Au/Ni shows meaningless hysteresis and RS behavior. It was assumed that a chemical change occurred in the Ag/SnSe during annealing. To confirm the effect of annealing on the I–V characteristics, the samples were annealed at different temperatures. After annealing at 120, 150, and 180 °C for 2 min, only high-temperature (>150 °C) annealing yielded hysteresis (Figure 3f). To confirm the chemical changes that occurred in Ag/SnSe, the SnSe interface before and after the annealing–peeling out process was measured with an energy−dispersive spectroscopy (EDS) line scan. The line−scan profile of the SnSe interface before annealing indicated Sn, Se, and O originating from native SnO_x_ (Figure 3g). However, the line−scan profile after the transfer–removal process showed the presence of Ag (Figure 3h). The presence of Ag indicates the diffusion of Ag ions. The same diffusion of Ag ions occurred after the evaporation of the Ag electrode. However, an important point here is that the LRS was observed only with excessive Ag ion transfer (Figure 3i). This also proves that the vdW integration induces stable RS behavior. The RS behavior occurred through a filament mechanism with Ag ions and e^−^ in the SnO_x_ layer (Figure 3j). Finally, Ni/SnSe/Ag memristors with vdW metal integration exhibited good performance. The performance of the memristor includes a low operating voltage for energy efficiency, reliability, on/off ratio, cycle stability, time variability, and temperature endurance. The low V_G_ was confirmed to be 0.4 and 0.1 V through the I–V curve by DC sweeping. Through the write (1 V)–read (0.1 V)–erase (−1 V)–read process, the memristor showed sufficient reliability and stability even at 4000 cycles. The resistance did not degrade after 10^5^ s, indicating the characteristics of nonvolatile memory. They demonstrated the potential of vdW integration deposition and a superior memristor with vdW metal integration.

#### 2.2.3. h−BN−Based Memristor

**Figure 4:**
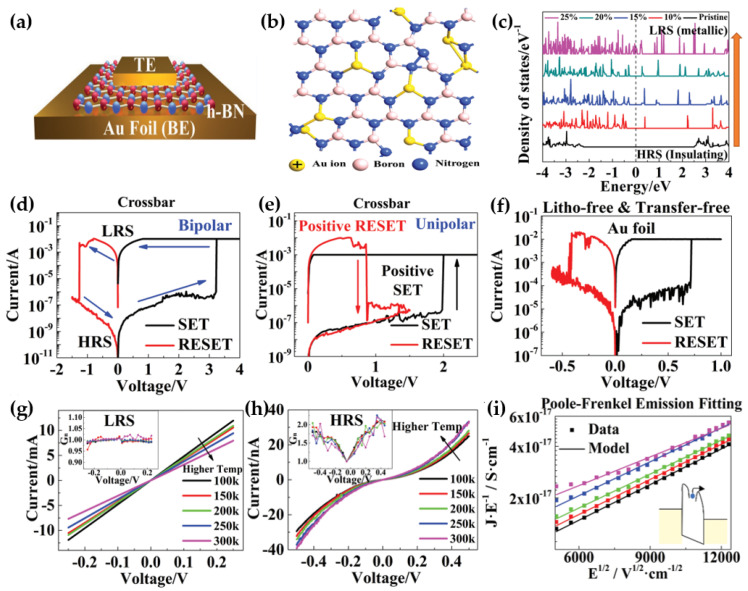
Monolayer h−BN is used in reported memristors. (**a**) Schematic of the h−BN MIM structure with Au top and bottom electrodes [47]. (**b**) Schematic showing boron vacancies replaced by Au ions [47]. (**c**) The density of states for boron vacancies is replaced by Au ions, with the dashed black line indicating the Fermi level of boron [47]. (**d**) Bipolar and (**e**) unipolar I–V curves measured at SET and RESET voltages in a crossbar array monolayer h−BN MIM device [47]. (**f**) Bipolar I–V curves of the monolayer h−BN MIM device fabricated on Au foil by chemical vapor deposition (CVD) [47]. (**g**,**h**) I–V curves for (**g**) LRS and (**h**) HRSs were measured on a crossbar array monolayer h−BN MIM device at various temperatures, with the inset showing normalized conductance (G_n_) [47]. (**i**) Fitting results of the HRS using Poole–Frenkel emission [47].

Wu et al. fabricated a monolayer hexagonal boron nitride (h−BN) memristor with nonvolatile resistance switching (NVRS) characteristics (Figure 4a) [47]. The device featured a MIM structure, with both the top and bottom electrodes composed of Au and the insulator layer made of a monolayer of h-BN. The resistance-switching mechanism in this device was attributed to the replacement of boron vacancies in h-BN by Au ions (Figure 4b) [48,49,50]. This substitution led to a reduction in the band gap and a decrease in resistance, as shown by the density of states for boron vacancies replaced by Au ions (Figure 4c). Initially, the device was in the HRS. By applying a positive bias of 3 V, the device transitioned into the LRS in the SET process, and no power was required to maintain this LRS. To switch back to the HRS, a negative bias of −1 V was applied during the RESET process (Figure 4d). Owing to the NVRS properties of h−BN, the device typically achieved an on/off ratio as high as 10^7^. Additionally, the unipolar I–V characteristics exhibited both SET and RESET processes at the same polarity as in the bipolar I–V curves (Figure 4e). The device’s performance was further enhanced through the fabrication of the MIM structure on Au foil using a low−pressure chemical vapor deposition (LPCVD) process. This lithography−free and transfer−free fabrication method prevented deterioration caused by residue (Figure 4f). The resistance switching behavior of the monolayer h−BN device was thoroughly examined, focusing on low−voltage read operations in both the LRS (Figure 4g) and HRS (Figure 4h). The LRS profile indicated that the electron transport mechanism had metallic characteristics, which were primarily attributed to the boron vacancies occupied by Au ions [51]. The normalized conductance of the LRS showed ohmic behavior. In contrast, the nonlinear I–V characteristics in the HRS increased with temperature (Figure 4h), suggesting a temperature−dependent transport mechanism. Among the various models evaluated, the Poole–Frenkel emission model was determined to be the most suitable for interpreting the HRS data. Consequently, the HRS data were fitted using this model (Figure 4i), with the results indicating that the boron vacancies in the monolayer h−BN acted as localized electron trapping centers during the HRS [52,53]. This study highlighted the advantages of using monolayer h−BN in memristors, specifically for achieving an ultrathin structure, a high on/off ratio, and minimal power consumption required to maintain the LRS. These properties demonstrate the effectiveness of monolayer h−BN for nonvolatile resistance switching applications, making it a promising material for future developments in this field.

#### 2.2.4. HfSe_2_−Based Memristor

**Figure 5:**
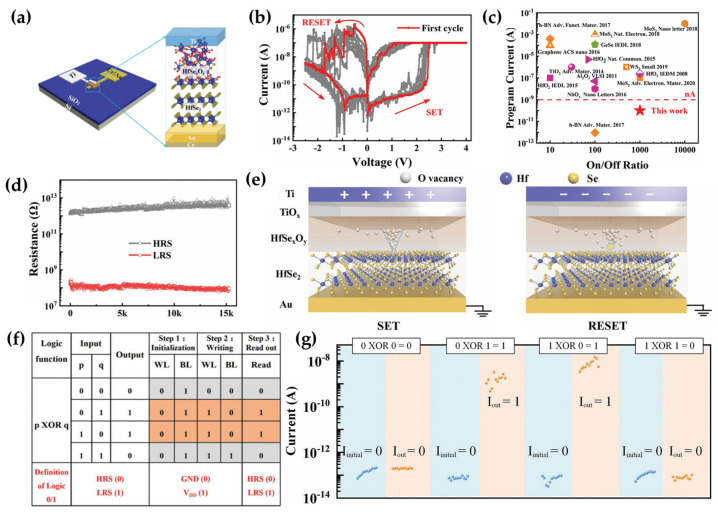
HfSe_2_ oxide−based energy−efficient logic memristor. (**a**) Schematic of the Ti/HfSe_x_O_y_/HfSe_2_/Au device structure [54]. (**b**) Repeatable bipolar resistive switching behavior at a low operating current of 100 nA [54]. (**c**) Comparison of the on/off ratio and operating current of this device with chalcogenides, transition metal oxides, and most 2D material−based memristors [54]. (**d**) Retention time demonstrating long−term stability over 15,000 s at 85 °C [54]. (**e**) Formation and rupture of cone-shaped filaments due to the concentration gradient of O and Se in the HfSe_x_O_y_ layer [54]. (**f**) Truth table of the XOR function shows four input combinations (p and q) [54]. (**g**) Logic results and operating current readout at a voltage of 0.1 V [54].

Liu introduced a hafnium diselenide (HfSe_2_) oxide−based low−power memristive logic device for Boolean computation, applicable to in−memory computing [54]. HfSe_x_O_y_ is oxidized from the HfSe_2_ layer by an O_2_ plasma treatment, inducing high resistance and a low operation current. The HfSe_2_ oxides are located between the top Ti electrode and the bottom Au electrode and serve as an RS medium (Figure 5a). The Ti/HfSe_x_O_y_/HfSe_2_/Au device exhibits repeatable bipolar RS behavior at a low 100 nA operation current (Figure 5b). Remarkably, even when the SET current decreases to 100 pA, significantly lower than the operational currents of memristors based on chalcogenides [55], transition metal oxides [56,57,58,59,60], and most 2D materials [49,51,61,62,63,64,65], the device maintains a large on/off ratio (Figure 5c). Furthermore, in this low−current mode, the device demonstrates excellent data retention (Figure 5d). The mechanism behind the RS behavior involves the formation of conductive filaments caused by oxygen vacancies. Titanium atoms extract oxygen from the HfSe_x_O_y_ layer, forming a thin Ti oxide layer between the Ti and HfSe_x_O_y_. The resulting oxygen vacancies generate the conductive filaments [56,66]. Due to the lower mobility of Se compared to O, fewer oxygen vacancies form near the HfSe_2_ layer, which has a higher concentration of Se. This difference in vacancy distribution results in a cone−shaped conductive filament, with more vacancies near the Ti electrode than near the HfSe_2_ layer [67]. The filaments are either formed or ruptured depending on the applied negative or positive bias (Figure 5e). To demonstrate the suitability of HfSe_2_−based memristors for low−power Boolean logic, the researchers employed a sequential logic method based on four variables [68,69]. The logic implementation consists of three steps. First, in the initialization step, the device is set to the HRS, which logically represents “0”, by applying 0 V and V_dd_ to the word line (WL) and bit line (BL), respectively. In the “write” step, XOR logic is used to assign the two input variables p and q to the BL and WL, with p = 0 for one configuration and the direction reversed for p = 1 (Figure 5f). The final resistance state of the memristor, representing the logic result, is then read using a read voltage of 0.1 V, yielding an ultralow current down to 100 pA (Figure 5g). In conclusion, this HfSe_2_−oxide−based memristor demonstrates low programming energy, making it a promising candidate for energy−efficient memory computing.

### 2.3. Hybrid Structure−Type 2D Material−Based Memristors

#### 2.3.1. rGO/GO/rGO−Based Memristor

**Figure 6:**
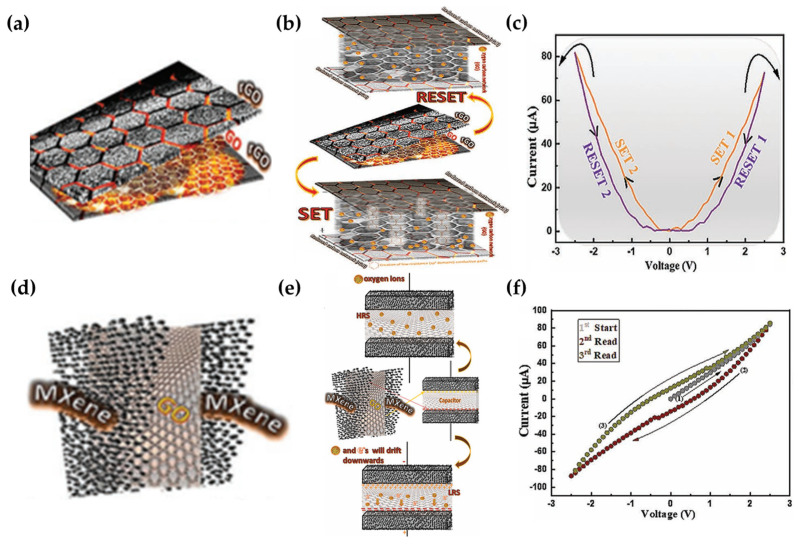
rGO/GO/rGO and M/GO/M memristors. (**a**) Schematic of the rGO/GO/rGO memristor [26]. (**b**) Schematic of RS behavior of the rGO/GO/rGO memristor [26]. (**c**) I–V characteristic of the rGO/GO/rGO memristor [26]. (**d**) Schematic of the M/GO/M memristor [26]. (**e**) Schematic of RS behavior of the M/GO/M memristor [26]. (**f**) I–V characteristic of the M/GO/M memristor [26].

Fatima et al. fabricated two types of memristors using reduced graphene oxide (rGO), graphene oxide (GO), and Ti_3_C_2_ MXene (M) [26,70,71,72,73,74,75]. The devices, rGO/GO/rGO and M/GO/M, were employed as RRAM (Figure 6a,d). The key to the RS behavior of these memristors is the GO layer, which contains oxygen ions and serves as the insulating medium. When a positive bias voltage is applied, oxygen defects within the GO layer migrate towards the top electrode. This migration leads to the transformation of the sp^3^ oxygen–carbon network in the GO layer into an sp^2^ carbon domain, forming conductive channels due to the oxygen vacancies (Figure 6b) [76]. The HRS transitions to the LRS via this vacancy migration mechanism (SET1 in Figure 6c). When the applied voltage is reduced from +2.5 V to 0 V, the memristor reverts to the HRS (RESET1 in Figure 6c). The RS behavior of the rGO/GO/rGO memristor is characterized by its I–V curve on a log–−log scale (Figure 6c). Ohmic conduction (I ∝ V) dominates in the HRSs, with slopes of 0.93 and 0.95, while space−charge limited current (SCLC) conduction (I ∝ V^2^) becomes dominant as the device switches from the HRS to LRS, with slopes of 1.63 and 2.46 [77,78]. This behavior suggests the activation of oxygen vacancies, oxygen holes, and charge traps within the GO layer during the HRS−to−LRS transition. Additionally, the I–V curve shows a hysteresis loop, indicating the potential for nonvolatile memory. During a positive DC sweep (0 V → +2.5 V → 0 V), the device undergoes a transition from SET 1 to RESET 1. Similarly, during a negative DC sweep (0 V → −2.5 V → 0 V), it transitions from SET 2 to RESET 2. In contrast, the M/GO/M memristor operates via a different mechanism compared to the rGO/GO/rGO memristor. Although a hysteresis loop is also observed in the I–V curve (Figure 6f), a non−zero interaction in the I–V curve is present, unlike in the rGO/GO/rGO device. The RS behavior of the M/GO/M memristor resembles that of a capacitive effect [79]. Positive and negative charge carriers, such as oxygen ions and electrons, accumulate at each electrode depending on the polarity of the applied voltage (Figure 6e). As a result, the memristor switches to the LRS. When 0 V is applied, the charge carriers neutralize, causing the device to revert to the HRS. However, a residual current remains at 0 V due to the presence of residual charge carriers (Figure 6f). The conductive mechanism of the M/GO/M memristor is further elucidated by the I–V curve on a log–−log scale. The device exhibits ohmic conduction with slopes of 0.96 and 0.40, followed by SCLC behavior with a slope of 1.93. A steep slope of 5.36 is also observed, representing a sharp increase in current transfer, which aligns with Child’s Law [80,81]. Furthermore, a hysteresis loop is present in the I–V curve. In Figure 6f, the device remains in the ON state for voltages between 0 V and +2.5 V (region 1). When the voltage exceeds +2.5 V, the device enters the write state. For voltages between +2.5 V and −2.5 V, the device enters the read state (region 2), and when the voltage surpasses −2.5 V, it transitions to the erase state. This behavior highlights the potential of these memristors for use in nonvolatile memory devices. Both the rGO/GO/rGO and M/GO/M memristors offer scalability, particularly in the absence of a substrate, which makes them suitable for flexible electrode applications [82]. Overall, carbon−based memristors demonstrate several advantages over conventional RRAM memristors.

#### 2.3.2. ZnO/WS_2_−Based Memristor

**Figure 7:**
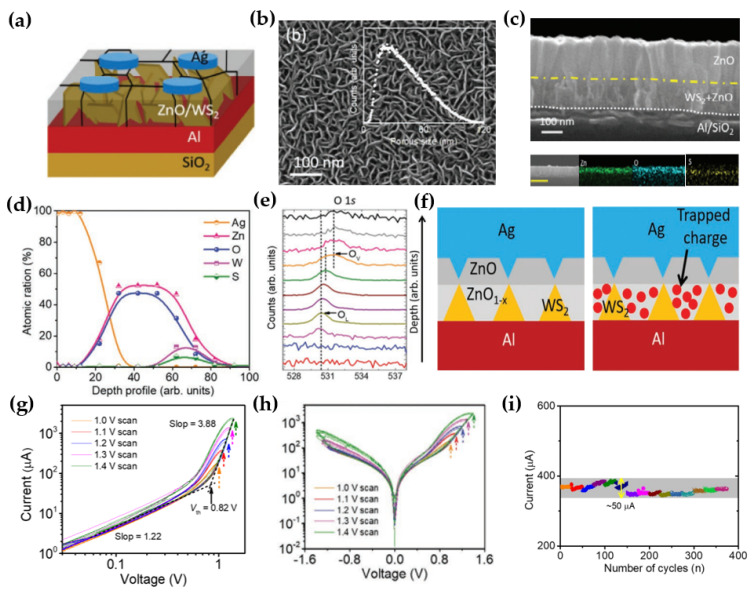
ZnO/WS_2_−based memristor. (**a**) Schematic of the ZnO/WS_2_ memristor [83]. (**b**) Planar SEM image of the WS_2_ layer [83]. (**c**) Cross−sectional scanning electron microscopy (SEM) and energy−dispersive spectroscopy (EDS) image of the ZnO/WS_2_ memristor [83]. (**d**) Atomic ratio vs. depth profile [83]. (**e**) O 1s depth−dependent X−ray photoelectron spectroscopy (XPS) spectra [83]. (**f**) Schematic illustrating charge trapping in the ZnO/WS_2_ memristor before and after trapping [83]. (**g**) I–V characteristics of the positive DC sweep in logarithmic scale [83]. (**h**) I–V curves in semi−log plots [83]. (**i**) Current measurements across 15 devices [83].

Kumar et al. fabricated a hybrid memristor that operates through a charge−trapping and de−trapping mechanism attributed to defective zinc oxide (ZnO) [83]. The memristor utilized Ag, Al, and a ZnO/WS_2_ heterostructure as the top electrode, bottom electrode, and resistive switching (RS) layer, respectively (Figure 7a). Metal oxide−based memristors typically exhibit RS behavior due to the local distribution of oxygen vacancies [63,84,85,86]. In this device, the defective ZnO grown using a WS_2_ layer provided enhanced control over the oxygen vacancy distribution, resulting in improved stability and reproducibility. The WS_2_ layer, grown on Al using a sputtering technique, created a porous medium with a vertical arrangement (Figure 7b). Within this porous structure, the defective ZnO served as the charge−trapping and de−trapping layer. The presence of Zn and O within the WS_2_ layer was confirmed via EDS mapping (Figure 7c) and further verified by depth−dependent X−ray photoelectron spectroscopy (XPS) (Figure 7d). In the XPS spectra of O 1s (Figure 7e), the peak shifted from approximately 530.6 eV to 531.8 eV, indicating the presence of oxygen vacancies, with the peak at 531.8 eV directly associated with the oxygen vacancy density. Before evaluating the device’s performance, the RS behavior was confirmed to be driven by charge trapping and de−trapping. The oxygen vacancy gradient in defective ZnO provided a platform for charge trapping (Figure 7f). This RS behavior was further validated using an I–V curve on a logarithmic scale, where a transition from low conductivity at a low bias (0 V to +0.82 V) to high conductivity at a higher bias (>0.82 V) demonstrated charge trapping (Figure 7g) [87]. Additionally, the temperature−dependent change in the I–V curve (Figure 7h) confirmed that the RS behavior was not due to a conductive filament mechanism [85,88,89]. The analog asymmetric hysteresis loop of the I–V curves indicated the potential application of this memristor in artificial synapses, such as for neuromorphic computing (Figure 7h) [90]. Furthermore, a wider hysteresis loop was observed with a larger DC sweeping range (±1.0 V to ±1.5 V), suggesting the possibility of programming the memristor at various current levels. A stable I–V curve was consistently obtained with repeated DC sweeps, indicating high device reproducibility. Additionally, the trap−filled limit voltage of +0.82 V remained constant under different conditions, further demonstrating the memristor’s cycle−to−cycle reproducibility. When 15 devices were tested over several cycles, the standard deviation of the current was only 11.83 µA, representing 3.18% of the average value, indicating high device−to−device reproducibility (Figure 7i). Overall, they demonstrated the potential of using a 2D RRAM memristor based on defective ZnO, induced by the porous characteristics of WS_2_, for charge trapping and de−trapping mechanisms.

#### 2.3.3. Graphene/h−BN/Graphen−Based Memristor

**Figure 8:**
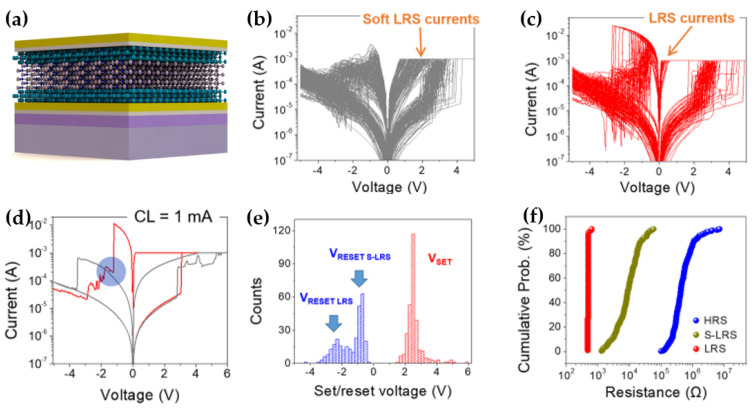
Graphene/hexagonal boron nitride/graphene stacked memristor demonstrating tristate RS operation. (**a**) The device structure of Au/Ti/G/h−BN/G/Au/Ti/SiO_2_/Si fabricated using chemical vapor deposition [91]. (**b**) I–V curves showing RS behavior between HRS and super−low−resistance state (S−LRS) [91]. (**c**) I–V curves between HRS and LRS [91]. (**d**) I–V curves illustrating RS among the three resistive states with current level = 1 mA [91]. (**e**) Distribution of V_set_ and V_reset_ [91]. (**f**) Cumulative probability of resistance values in HRS, S−LRS, and LRS [91].

Zhu et al. introduced the first 2D material−based memristor capable of exhibiting three distinct resistive switching levels, including a soft −low resistive state (S−LRS), which lies between the HRS and LRS [91]. To enhance memristor performance, 2D materials were integrated into a metal/transition metal oxide/metal structure [92,93]. The authors fabricated a 5 µm × 5 µm cross−point Au/Ti/graphene (G)/hexagonal boron nitride (h−BN)/G/Au memristor (Figure 8a), which demonstrated switching to either a more conductive or less conductive state by modulating the current level or reset voltage, respectively. The h−BN stack contained conductive regions associated with defects, which are essential for RS behavior. The device switched between the HRS and S−LRS when a current level of 0.5 mA was applied (Figure 8b) and between the HRS and LRS when a current level of 5 mA was applied (Figure 8c). Notably, at a current level of 1 mA, the device exhibited switching between three resistive states, a metastable RS behavior not previously observed in any 2D material−based memristor (Figure 8d). The variability of the set (V_set_) and reset (V_reset_) voltages further demonstrated the applicability of this device to memristive technologies (Figure 8e). Additionally, there was no overlap between the three resistive states, indicating that they are distinguishable (Figure 8f). Notably, larger devices, with dimensions of 100 µm × 100 µm and the same Au/Ti/G/h−BN/G/Au stacked structure, did not exhibit the metastable tri−state RS behavior [49]. This discrepancy is attributed to the larger device size, which is comparable to the grain size of the copper foil used for h−BN growth [94], leading to more defects along the grain boundaries that prevent S−LRS formation. Moreover, 5 µm × 5 µm devices without graphene in the structure showed similar results. This is because graphene acts as a barrier, restricting ion exchange between the metal and h−BN. Consequently, the formation of conductive nanofilaments (CNFs) in defect−free regions of the 2D films and the size of these CNFs play a crucial role in S−LRS formation.

#### 2.3.4. Robust Graphene/MoS_2−x_O_x_/Graphene (GMG)−Based Memristor

**Figure 9:**
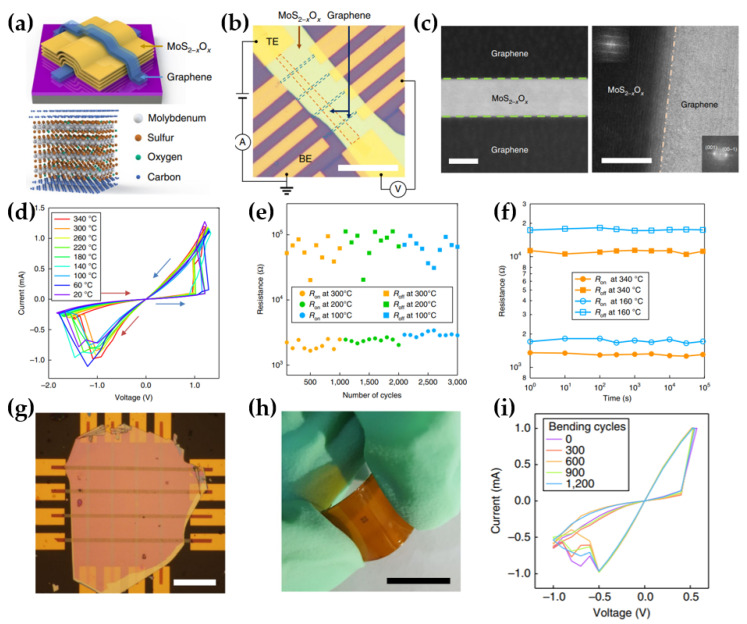
Graphene/MoS_2−x_O_x_/graphene (GMG) memristor. (**a**) GMG memristor image. Top: schematic of the GMG device. Bottom: Three−dimensional image of the GMG device’s graphene/MoS_2−x_O_x_/graphene stack [63]. (**b**) Optical image of the GMG device (scale bar: 25 μm). The highlighted blue and orange dashed boxes are the bottom and top graphene electrodes, respectively [63]. (**c**) Cross−sectional HAADF image (left) and HRTEM image (right) of the GMG device (scale bar: 20 nm) [63]. (**d**) I–V Characteristic of the GMG device at various temperatures. Switching directions are indicated by arrows [63]. (**e**) I–V curves performed 1000 cycles at 300 °C (orange), 200 °C (green), and 100 °C (blue) under 1 μs pulse [63]. (**f**) Retention time of the GMG device at 340 °C (orange) and 160 °C (blue). Resistance measured at V_r_ = 0.1 V [63]. (**g**) Optical microscope image of the GMG device fabricated crossbar on PI substrate (scale bar: 20 μm) [63]. (**h**) Image of the crossbar−type GMG device under bending (scale bar: 10 mm). (**i**) I–V curve of the mechanically bent GMG device (1 cm radius curvature) [63].

Wang et al. fabricated a highly durable memristor using a 2D material stack of graphene/MoS_2_−_x_O_x_/graphene (GMG) [63]. The GMG device was constructed with graphene as both the top and bottom electrodes (TE and BE, respectively) (Figure 9a). After mechanically exfoliating multilayer graphene and MoS_2_, the materials were transferred onto SiO_2_/Si wafers. The graphene electrodes and MoS_2−x_O_x_ layer formed vdW bonds, which contributed to the device’s thermal stability. The switching behavior of the GMG device was attributed to the increased resistance caused by the replacement of sulfur vacancies with oxygen vacancies in the MoS_2−x_O_x_ layer. The sulfur vacancies were generated by the thermophoresis effect [95,96], resulting from ohmic heating produced by the current flowing in the conductor. The temperature gradient created by the increasing current displaced sulfur ions from the MoS_2−x_O_x_ layer. An optical image of the GMG device and its measurement setup revealed a structure stacked via vdW heterostructures, which were formed using the polyvinyl alcohol−based transfer method (Figure 9b). Four−probe measurements were employed to eliminate the resistance contribution from the graphene electrodes. The performance of a memristor device can vary significantly depending on the roughness of its interface [97,98]. In Figure 9c, high−angle annular dark field (HAADF) and high−resolution transmission electron microscopy (HRTEM) images reveal the MoS_2−x_O_x_ layer formed by vdW heterostructures, oxidized at 160 °C for 1.5 h. vdW heterostructures combine the excellent electrical properties and structural stability of each 2D material component through atomically sharp interfaces [99,100].

The GMG device exhibited repeatable bipolar switching characteristics enabled by vdW heterostructures and high−temperature electroforming. The I–V curves of GMG devices were recorded at temperatures ranging from 20 to 340 °C (Figure 9d). The device operated reliably even at a high temperature of 340 °C, demonstrating excellent thermal stability and maintaining performance without degradation at elevated temperatures. The stable switching window and cycle−to−cycle reproducibility of the GMG device were well −maintained at the ON and OFF states across three different temperatures, 100, 200, and 300 °C, as the temperature increased (Figure 9e). Additionally, the resistance change and retention time in both the ON and OFF states remained stable for up to 10^5^ s, exhibiting high reliability even at elevated temperatures (Figure 9f). Both MoS_2_ and graphene possess excellent mechanical flexibility [101,102], making them ideal for robust electronics designed for flexible applications under mechanical stress [103,104]. To demonstrate this flexibility, a crossbar−shaped GMG device was fabricated on a polyimide (PI) substrate (Figure 9g,h). The I–V sweeps of the GMG device, performed under bending conditions (strain of 0.6%), revealed stable switching curves even after 1200 bending cycles (Figure 9i). This performance indicates that the device maintained a consistent state and exhibited excellent mechanical durability. In conclusion, the robust memristor, based on graphene/MoS_2_−_x_O_x_/graphene vdW heterostructures, demonstrated high endurance even in harsh environments such as high temperatures and mechanical bending conditions.

### 2.4. Two−DimensionalMaterial−Based Memristors for Neuromorphic Computing

#### 2.4.1. PdSeO_x_/PdSe_2_−Based Memristor

**Figure 10:**
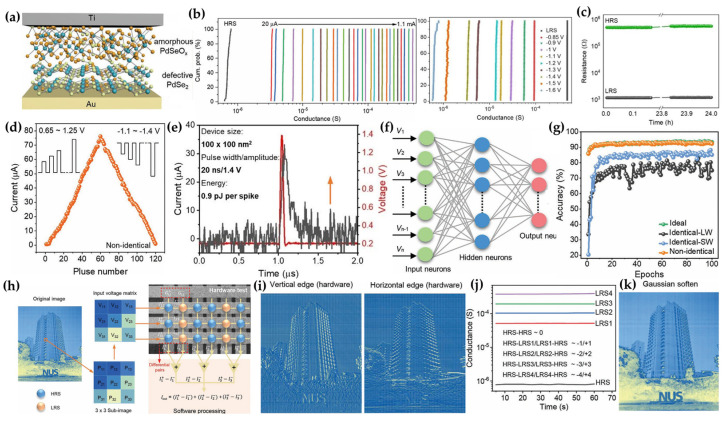
Application of the PdSeO_x_/PdSe_2_ memristor. (**a**) Schematic illustration of the PdSeO_x_/PdSe_2_ memristor [105]. (**b**) Representation of the multilevel LRS and HRS multilevel [105]. (**c**) Resistance versus time plot demonstrating retention [105]. (**d**) Long−term depression (LTD) and long−term potentiation (LTP) processes with pulse amplitudes ranging from 0.65 V to 1.25 V in 0.01 V increments for LTP and from −1.1 V to −1.4 V in 0.005 V increments for LTD. The pulse period and width are fixed at 5 ms and 0.1 ms, respectively [105]. (**e**) Switching operation using a 20 ns pulse [105]. (**f**) Schematic of a three−layer artificial neural network (ANN) [105]. (**g**) Image recognition accuracy as a function of training epochs [105]. (**h**) Convolutional image processing using the memristor crossbar array [105]. (**i**) Vertical and horizontal edge detection in hardware processing [105]. (**j**) Illustration of five distinct multilevel resistance states [105]. (**k**) Gaussian−blurred image produced using five multilevel resistance states [105].

Li et al. demonstrated a high image recognition accuracy of 93.40% using ultrathin PdSeO_x_/palladium diselenide (PdSe_2_) memristors [105]. The hybrid insulator layer (PdSeO_x_/PdSe_2_) of this memristor was formed through transformation rather than deposition. The top layer of intrinsic PdSe_2_ was converted to PdSeO_x_ using UV–ozone treatment (Figure 10a). This treatment enabled the memristor to exhibit ultrathin characteristics and high reliability. For building artificial neural networks (ANNs) [106], multilevel switching and retention are critical properties. The PdSeO_x_/PdSe_2_ memristor demonstrated multilevel resistive switching states and high retention, making it highly promising for neuromorphic computing applications (Figure 10b,c). Synaptic plasticity, which underpins neuromorphic computing, was achieved through pulse−induced switching. The research focused on long−term synaptic plasticity for neuromorphic computing, where linearity in plasticity is essential for ANN functionality. Although nonlinear−long−term plasticity was observed using identical pulses, linear and symmetric long−term plasticity could be achieved with non−identical pulses (inset in Figure 10d), as shown in Figure 10d. Additionally, the memristor exhibited low energy consumption (0.9 pJ) with short pulses of 20 ns (Figure 10e), which is comparable to the energy efficiency of biological synapses in the brain [107]. The ANN of PdSeO_x_/PdSe_2_ memristors was simulated using the NeuroSim+ platform, with images from the Modified National Institute of Standards and Technology (MNIST) dataset for training and recognition [106]. The ANNs consisted of 400 input neurons, 100 hidden neurons, and 10 output neurons (Figure 10f). Numbers 0–9 were registered in the output neurons, implemented with 20 × 20 pixels, and trained using approximately 70,000 images from MNIST. The recognition accuracy with 10,000 images not used in training showed a high accuracy of 93.40%, comparable to the ideal device accuracy of 93.90% (Figure 10g). The researchers also applied convolutional image processing (CIP) using a crossbar array memristor. Each pixel of the input image (NUS building) was generated by convoluting the conductance vectors (G_ij_) and input voltage (V_ij_), mapped from the conductance matrix and 3 × 3 input subimage, respectively (Figure 10h). The currents I^+^ and I^−^, calculated as ΣV_ij_ × ΣJ_ij_, were implemented using the crossbar array through Ohm’s law and Kirchhoff’s current law [108]. The total output current I_out_ (I^+^ + I^−^) was converted to pixel (P_out_). Thus, the vertical and horizontal edge images were successfully extracted. (Figure 10i). Furthermore, with weights ranging from −4 to +4, derived from the retention of five multilevel resistive states (Figure 10j), a complex convolution kernel, such as Gaussian softening, was constructed (Figure 10k). The ultrathin PdSeO_x_/PdSe_2_ memristors exhibited excellent performance and demonstrated significant potential for various applications, including ANN and convolutional image processing.

#### 2.4.2. Wafer−Scale MoS_2_−Based Memristor

**Figure 11:**
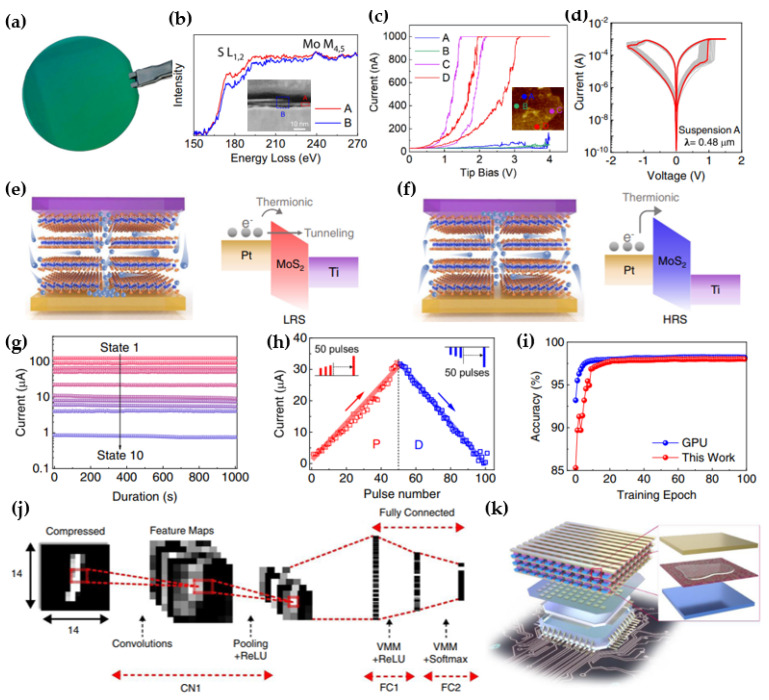
Applications of wafer−scale MoS_2_−based Pt/MoS_2_/Ti memristors. (**a**) Wafer−scale MoS_2_ film [109]. (**b**) Electron energy loss spectroscopy (EELS) spectra of parts A and B from the inset. The inset shows the cross−sectional annular dark−field scanning transmission electron microscope (ADF−STEM) images of the MoS_2_ film [109]. (**c**) I–V characteristics were measured using C−AFM. The inset displays an atomic force microscopy (AFM) image of the MoS_2_ flake, where A and B represent the central parts and C and D denote the edges [109]. (**d**) I–V curve of the memristor with a size of 0.48 µm [109]. (**e**,**f**) Schematics illustrating the (**e**) set and (**f**) reset processes of the conduction mechanism [109]. (**g**) Multilevel resistive states of MoS_2_ memristors [109]. (**h**) Long−term potentiation (LTP) and long−term depression (LTD) processes (pulse width: 100 ns; LTP pulse amplitudes: 1.0 V to 1.75 V in 15 mV steps; LTD pulse amplitudes: −1.5 V to −2.25 V in 15 mV steps, pulse width: 500 ns) [109]. (**i**) Image recognition accuracy as a function of the training epoch [109]. (**j**) Schematic of the three−layer deep CNN, where a 14 × 14 compressed image is derived from a 28 × 28 original image [109]. (**k**) Schematic diagram of the 3D memristor [109].

Tang et al. demonstrated multiple applications of Pt/MoS_2_/Ti memristors [109]. They fabricated wafer−scale MoS_2_ on a 2−inch Si/SiO_2_ substrate using MoS_2_ suspension and the spin−coating method (Figure 11a). This approach resulted in a memristor with a high yield. The MoS_2_ sheet exhibited weak intensity at the edges (S−L_1,2_) (Figure 11b), indicating that sulfur vacancies were distributed along the flake edges. To confirm this observation, measurements were conducted on the central regions (A and B in Figure 11c) and edges (C and D in Figure 11c) using C−AFM. A hysteresis window was evident in the I–V curves of the edges (C and D), while no such window was observed in the I–V curves of the central regions (A and B) (Figure 11c). This further confirmed that sulfur vacancies, playing a significant role in the conduction mechanism of the memristor, were concentrated at the edges. As the size of the device decreases, the ratio of edges increases, thereby affecting the performance of the memristor. I–V measurements conducted on memristors of various sizes (λ) revealed that the 0.48 µm memristor exhibited the lowest V_set_/V_reset_ values (0.65 V and −0.90 V) compared to those with larger sizes of 1.20 and 2.40 µm (Figure 11d). The conduction mechanism of this memristor is illustrated in Figure 11e,f. Upon applying a positive bias to the top electrode (Pt), the sulfur vacancies from the edges migrate toward the bottom electrode (Ti). This process results in the lowering of the Schottky barrier, facilitating the transition from the HRS to the LRS (Figure 11e). Conversely, when a negative bias is applied, the sulfur vacancies return to the edge (Figure 11f), causing the conductive filaments to rupture and transitioning the LRS back to the HRS. The Pt/MoS_2_/Ti memristor demonstrated robust and reliable performance, maintaining on/off states for 1 × 10^5^ s without degradation and sustaining 1 × 10^7^ cycles. Additionally, the memristor exhibited multilevel resistance states for single−device programming (Figure 11g). For a three−layer convolutional neural network (CNN), symmetric and linear synaptic long−term plasticity was achieved (Figure 11h) [110,111,112]. After training a three−layer deep CNN on MoS_2_ RRAM with 50,000 images, a recognition accuracy of 98.02% was attained (Figure 11i). Furthermore, a 3D MoS_2_ memristor−based monolithic 3D circuit was proposed to minimize crosstalk issues and accurately program each memory cell (Figure 11j) [113,114]. These findings highlight the advantages of MoS_2_ on a wafer scale and the potential for various applications of 2D material−based memristors.

## 3. Summary and Challenges

The 2D materials examined in this study, offering low power consumption, ultra−thin film, and excellent electrical and physical properties, are ideal for next−generation neuromorphic computing. As previously highlighted, the development of high−efficiency artificial synaptic devices and brain simulation results provides solutions for achieving advanced neuromorphic computing. However, as shown in Table 1, despite the excellent memristive properties, progressing toward neuromorphic computing applications remains a significant challenge. Two−dimensional materials still encounter various challenges that must be addressed during the initial research phase. Large−area and high−density integration is essential for emulating the human brain; however, chip−level experimental validation remains insufficient. Chemical vapor deposition (CVD) is essential for the large−area deposition of 2D materials. However, CVD−deposited 2D materials introduce several uncertainties, such as uneven thickness, defects, and variations in grain size. These issues result in reduced uniformity and reproducibility across different devices, which in turn degrade the uniformity and reproducibility of neuromorphic computing applications. Recently, successful demonstrations of large−area 2D material film formation and thickness reproducibility using CVD by various research groups have begun to address deficiencies in this area [115,116]. Nonetheless, some existing difficulties must still be resolved. Concerning physical properties and doping strategies, conventional ion implantation processes are inadequate for inducing doping in 2D materials at the atomic level; thus, alternative doping methods must be investigated, including gas−induced doping techniques. Additionally, because 2D materials consist of atomic units, these devices are prone to degradation due to reactions and oxidation in surrounding environments or atmospheres. This paper proposes a protective layer−forming process to mitigate this issue. Furthermore, extremely small patterning technologies are necessary to avoid compromising the original switching performance and artificial synapse functionality, which can lead to instability in the SET and RESET voltages of memristors, deviations between devices, and significant cycle−to−cycle variations. The underlying factors governing the randomness associated with the formation and extinction of conductive pathways in 2D materials remain unclear, and studies aimed at reliably inducing these phenomena should be conducted. From an industrial perspective, challenges such as compatibility with existing CMOS technology, yield rates, and mechanical stability remain significant. These difficulties arise from the high processing temperatures needed for synthesis, the requirement for uniform thickness over large−scale areas, and the extremely thin nature of 2D materials. Therefore, to construct next−generation artificial neural networks, ongoing research on 2D materials is essential [117]. However, despite the numerous challenges, it is important to emphasize that the potential benefits outweigh the disadvantages, and the successful construction of artificial neural networks will be feasible through architectural studies and innovative design methods that address these issues.

## Figures and Tables

**Table 1:** Performance metrics of 2D material−based memristors of this paper.

Material	Switching Type	On/Off Ratio	SET and RESET Voltage	Endurance	Retention Time	Switching Speed	Recognition Accuracy	Ref.
α−In_2_Se_3_	Digital	>10^3^	SET: 3 V RESET: −3 V	100 cycles	>5000 s	10 ns	93.20%	[32]
SnSe	Digital	>10^3^	SET: 0.4 V RESET: −0.1 V	4000 cycles	10^5^ s	−	−	[36]
h−BN	Digital	>10^7^	−	>50 cycles	>10^5^ s	<15 ns	−	[42]
HfSe_2_	Digital	10^3^	SET: 2.32 V RESET: −0.7 V	>40 cycles	15,000 s	<50 ns	−	[49]
Graphene oxide	Analog	−	−	2400 cycles	10⁴ s	−	−	[21]
Graphene oxide	Analog	−	−	2400 cycles	−	−	−	[21]
ZnO/WS_2_	Analog	300 by 100 pulses	−	10^4^ cycles	−	−	−	[79]
h−BN	Digital	> 10	SET: 2.5 V RESET: −1 V	100 cycles	−	−	−	[87]
MoS_2−x_O_x_	Digital	10	SET: 3 V RESET: −4 V	10^7^ cycles	10^5^ s	<100 ns	−	[59]
PdSeO_x_/PdSe_2_	Digital	~10^2^	SET: 0.58 V RESET: −0.71 V	>700 cycles	>80,000 s	−	93.19%	[101]
MoS_2_	Digital	>10	SET: 3 V RESET: −4 V	10^7^ cycles	10^5^ s	−	98.02%	[105]
